# Development and Characterization of Composites Consisting of Calcium Phosphate Cements and Mesoporous Bioactive Glass for Extrusion-Based Fabrication

**DOI:** 10.3390/ma12122022

**Published:** 2019-06-24

**Authors:** Richard Frank Richter, Tilman Ahlfeld, Michael Gelinsky, Anja Lode

**Affiliations:** Centre for Translational Bone, Joint and Soft Tissue Research, University Hospital Carl Gustav Carus and Faculty of Medicine, Technische Universität Dresden, 01307 Dresden, Germany; richard.f@rfrport.net (R.F.R.); tilman.ahlfeld@tu-dresden.de (T.A.)

**Keywords:** calcium phosphate cement, mesoporous bioactive glass, additive manufacturing, 3D plotting, strontium ion release

## Abstract

Calcium phosphate cements (CPC) and mesoporous bioactive glasses (MBG) are two degradable biomaterial groups widely under investigation concerning their applicability to treat bone defects. MBG-CPC composites were recently shown to possess enhanced degradation properties in comparison to pure CPC. In addition, modification of MBG allows an easy incorporation of therapeutically effective ions. Additive manufacturing of such composites enables the fabrication of patient-specific geometries with further improved degradation behavior due to control over macroporosity. In this study, we developed composites prepared from a non-aqueous carrier-liquid (cl) based CPC paste and MBG particles suitable for extrusion-based additive manufacturing (3D plotting). CPC with the addition of up to 10 wt % MBG were processible by adjusting the amount of cl. Scaffolds consisting of a 4, 6 and 8%-MBG-CPC composite were successfully manufactured by 3D plotting. While mechanically characterization of the scaffolds showed an influence of the MBG, no changes of microstructure were observed. During degradation of the composite, the release of Ca^2+^ and Sr^2+^ ions could be controlled by the MBG composition and plotted scaffolds with macropores showed a significant higher release than bulk samples of comparable mass. These findings demonstrate a high flexibility regarding ion release of the developed composites and suggest utilizing the drug binding capacities of MBG as a prospective delivery system for biologically active proteins.

## 1. Introduction

Calcium phosphates (CaP) are ceramic biomaterials suitable for treatment of bone defects. Several studies have shown the benefit of soluble and insoluble CaP phases for osteogenesis in vitro and in vivo [1,2,3]. A special class of CaP are pasty calcium phosphate cements (CPC), which are a suspension of a liquid phase and at least one CaP precursor in a liquid phase [4]. In case of CaP precursors with hydraulic reactivity, like α-tricalcium phosphate (α-TCP), a self-induced dissolution-precipitation reaction chain starts immediately in contact with aqueous media with the formation of non-stochiometric calcium-deficient hydroxyapatite (CDHA). Those self-setting CPC formulations traditionally get prepared within the operating theater by mixing the precursor powder with an aqueous liquid and have to be injected into injured bone tissue quickly. Otherwise the precipitation reaction will hinder extrudability of the cement through a needle, caused by a filter-pressing effect, which describes the occurrence of already set CaP precipitates within the needle leading to phase separation and needle-clogging [5]. Therefore, several studies investigated the possibility to retard the setting reaction of the precursors of calcium phosphate cements [6,7,8,9]. To specifically enhance the injectability of α-TCP-based CPC formulations, polymers like gelatin [10] or Pluronic F127 [11] or a water-immiscible, biocompatible oil (acting as temporary carrier liquid (cl) which gets exchanged with water when setting of the CPC occurs) [12] were introduced into the formulation. These CPC formulations retarded the setting reactions to a great extent and were shown not only to allow easy extrusion through syringes, but also to be applicable for low-temperature extrusion-based additive manufacturing (called robocasting or 3D plotting) [13,14,15].

In contrast to conventional powder–liquid systems, the cl-based CPC used in this study possesses a long storability and processability, which not only results in a great reproducibility, but makes it also highly suitable for 3D plotting [12,13]. Besides, it was shown, that biologically active growth factors can be incorporated into the CPC formulation by addition of growth factor-laden chitosan/dextran sulphate microparticles before fabrication [16]. It was found that setting in water-saturated atmosphere (humidity > 95%) for 3 d was appropriate for phase transformation from α-TCP to CHDA while preventing loss of the loaded growth factor during setting and maintaining of its biological activity. Moreover, this post-processing of the CPC scaffolds avoided formation of microcracks which impaired the mechanical stiffness of CDHA scaffolds significantly [16]. Furthermore, it was shown that by using multichannel plotting, the pasty CPC can be combined with other materials such as hydrogels (loaded with growth factors or even cells) which allows the fabrication of complex scaffolds [17,18].

Schumacher et al. showed recently the development of a mesoporous bioactive glass (MBG)—CPC composite for controlled growth factor delivery [19]. The incorporation of MBG particles in a powder–liquid cement led to an facilitated sample degradation and therefore higher porosity after 21 days of immersion in water [19]. In addition, the composite showed promising in vitro results regarding its cytocompatibility and release properties for proteins which were loaded onto MBG particles prior to mixing with the CPC [19]. Later, a non-aqueous carrier-liquid (cl) based CPC formulation was used in order to circumvent the limited processing time of the powder–liquid system. Kauschke et al. investigated such a composite in vivo in a murine fracture model and observed 35 days after implantation pores within the composites that were formed by dissolution of MBG particles and filled with newly formed bone tissue [20]. However, until now the extrusion properties of the cl-based MBG-CPC composites have not been studied in detail. Herein, we investigated the extrudability of MBG-CPC composites with different MBG amounts by measuring the mass flows through conical plotting needles of various inner needle diameters (250, 410, 610 µm). To easily fabricate MBG-containing CaP scaffolds by 3D plotting, we optimized the system by adjusting the amount of cl within the CPC formulation. Afterwards, MBG-CPC scaffolds were fabricated by 3D plotting, set in humidity and porosity, microstructure and mechanical properties were characterized. Finally, MBG containing strontium ions, which were shown to promote osteogenesis, were fabricated and mixed with CPC. The release of calcium and strontium ions from plotted scaffolds was investigated in comparison to bulk samples. This manuscript shows the development of a highly flexible MBG-CPC composite material system which can be used to tailor the release of ions and which will be applicable for an enhanced release of growth factors in future.

## 2. Materials and Methods

### 2.1. Synthesis and Characterization of MBG

MBG powders were synthesized according to the method proposed by Yan et al. [21]. In a typical synthesis for the basic MBG (molar ratio of Si/Ca/P = 80:15:5), 4.0 g of the non-ionic block polymer Pluronic P123 (molecular weight Mw = 5800, Sigma-Aldrich, Steinheim, Germany) were dissolved in 60 g of ethanol (96%) and stirred at room temperature for 1 h. Next, 6.7 g of tetraethyl orthosilicate (99%, Sigma-Aldrich, Steinheim, Germany), 0.73 g of triethyl phosphate (99.8%, Sigma-Aldrich, St. Louis, MO, USA), 1.4 g of calcium nitrate-tetrahydrate (dissolved in 6 mL deionized water, Merck, Darmstadt, Germany) and 1.0 g of 0.5 M HCl were added. The complete solution was stirred at room temperature for 24 h and casted afterwards in petri dishes left for drying at room temperature for 1 d to undergo an evaporation-induced self-assembly process. The obtained gel was dried at 60 °C for 3 h, grinded and then calcined at 700 °C for 7 h. Obtained glass particles were manually grinded and fractionated by vibration sieving with 500, 250, 180 and 45 µm sieves. Strontium-containing MBG were produced in the same way by gradually substitution of the calcium by strontium [22], using strontium nitrate (Sigma-Aldrich, Steinheim, Germany) as Sr^2+^ source. Due to the lower solubility, Sr(NO_3_)_2_ was dissolved beforehand in 20 mL deionized water.

### 2.2. Synthesis and Characterization of MBG-CPC Composites

#### 2.2.1. Preparation of MBG-CPC Composites

Ready-to-use CPC and carrier liquid (cl) were obtained from INNOTERE GmbH (Radebeul, Germany); compositions of both components were as recently described [23]. MBG-CPC composites were prepared by thoroughly manual mixing of the components. Based on the amount of CPC paste, parts of MBG [w_MBG_/w_CPC_ %] and carrier liquid [w_cl_/w_CPC_ %] were added, respectively. An overview of all prepared compositions is shown in Table 1.

#### 2.2.2. Injectability and Extrusion Properties

Extrusion properties of MBG-CPC composites were evaluated by measuring the mass flow through conical plotting needles (Globaco, Rödermark, Germany). Therefore, plotting cartridges (Nordson EFD, Oberhaching, Germany) were filled with similar volumes of MBG-CPC pastes and different air pressures (100, 200, 300, 400, 500 kPa) were applied for 20 s, subsequently the extruded mass was measured with a precision scale. CPC and X%-MBG-CPC (X = 1, 2, 3, 4) were tested for inner needle diameters of 610, 410 and 250 µm and composites with higher MBG amounts (X = 5, 6, 7, 8, 10) were tested with an inner needle diameter of 610 µm. Storability of MBG-CPC pastes was determined by measuring the mass flow of freshly prepared 2%-MBG-CPC, repeating the measurement after 3 d and 7 d and comparing it to pure CPC.

#### 2.2.3. Setting Time

Setting properties were examined by the Gillmore-needle-test (weight = 113.4 g, needle ø = 2.13 mm). Cylindrical samples (ø = 12 mm, h = 7 mm, n = 16) of pure CPC, CPC-cl and 8%-MBG-CPC were set in water-saturated environment (humidity > 95%) at 37 °C and initial setting time (t_i_) was measured (measuring intervals: 15 min until first sample reached t_i_, 5 min afterwards).

### 2.3. Scaffold Fabrication by 3D Plotting

#### 2.3.1. Scaffold Design, Plotting Procedure and Post-Processing

Plotted scaffolds were fabricated using a multichannel 3D plotter (Bioscaffolder 2.1, GeSiM mbH, Radeberg, Germany). Scaffolds (12 mm × 12 mm) with a layer-to-layer-orientation of 90°, a layer thickness of 0.3 mm and a strand distance of 1.2 mm were plotted with a 410 µm needle. The plotting parameters for different material compositions are shown in Table 2. Subsequent to plotting, scaffolds were incubated in water-saturated atmosphere at 37 °C (humidity > 95%) for 3 d. 

#### 2.3.2. Porosity Measurement

Porosity of plotted scaffolds was determined by helium-pycnometry (Ultrapyc 1200e, Quantachrome). The porosity (P) was calculated from the quotient of apparent volume (V_app_) and obtained pycnometric volume (V_pyc_) according to the following equation:

(1) $$P=\;\frac{V_{\mathrm{app}}-{\;V}_{\mathrm{pyc}}}{V_{\mathrm{app}}}\;\times 100\%$$

#### 2.3.3. Microscopy

Macroporosity and shape fidelity of plotted scaffolds were investigated by stereo microscopy (Leica M205C (Leica, Wetzlar, Germany) equipped with a DFC295 camera, Leica, Wetzlar, Germany). For microstructure analysis, scaffolds were coated with carbon, sputtered with gold and analysed using scanning electron microscopy (SEM; JEOL JGM-7500F Field emission microscope, JOEL, Freising, Germany).

#### 2.3.4. Mechanical Characterization

Plotted cubic-shaped scaffolds (12 mm × 12 mm, h = 3 cm; n = 5) of CPC as well as 4%-, 6%- and 8%-MBG-CPC were set in water-saturated atmosphere for 3 d and incubated subsequently in deionized water for a further 7 d. Afterwards, uniaxial compressive tests were performed with a speed of 1 mm⋅min^−1^ using a Zwick universal testing machine (Z010 equipped with a 10 kN load cell, Zwick, Ulm, Germany). Compressive modulus and compressive strength were obtained from the data.

### 2.4. Release of Calcium and Strontium Ions

Plotted scaffolds (n = 6) consisting of an 8%-MBG-CPC paste containing MBG of different Sr content (0, 5, 10, 15 mol %) and bulk scaffolds (ø = 13 mm, h = 1 mm, n = 6) consisting of the 8%-15SrMBG-CPC composite were fabricated. All samples were set in water-saturated atmosphere for 3 days at 37 °C, washed in acetone, air-dried and weighted afterwards. Then, each sample was incubated in 2 mL phosphate-buffered saline (PBS) and treated under cell culture conditions. After 1, 4, 7, 10, 14, 18 and 21 d, supernatants were exchanged completely with fresh PBS; the harvested supernatants were frozen at −80 °C. Calcium and strontium ion content within thawed supernatants were analysed using inductively coupled plasma-optical emission spectroscopy (ICP-OES, Plasma Quant PQ 9000 Elite, Analytik Jena, Jena, Germany). Therefore, 1 mL of supernatant was mixed with 65% nitric acid and filled up with deionized water to a final acid concentration of 2%. The obtained calcium and strontium ion concentrations were normalized to the measured weight of the scaffolds.

### 2.5. Statistical Evaluation

One-way analysis of variance (ANOVA), followed by a multiple comparison test with Turkey test was performed to determine statistically significant differences of the obtained data. Statistical significance was assessed at *p* < 0.05.

## 3. Results

### 3.1. Preparation of Mesopouros Bioactive Glass (MBG) Particles

To obtain MBG powder consisting of various particle sizes, MBG were produced as recently described [21], manually grinded and fractionated by vibration sieving. After grinding, more than 50% of MBG particles revealed an average diameter >180 µm (Figure S1A). Therefore, a second manual grinding step was performed; then, more than 50% of the MBG particles had an average diameter between 180 and 45 µm. After a third grinding step, more than 99% could be identified to be smaller than 45 µm; this fraction tended to agglomerate while vibration (Figure S1B), but the MBG particles could be separated again by gentle movement. Hence, these particles were used for subsequent experiments.

### 3.2. Storability of MBG-CPC Composite Pastes

**Figure 1:**
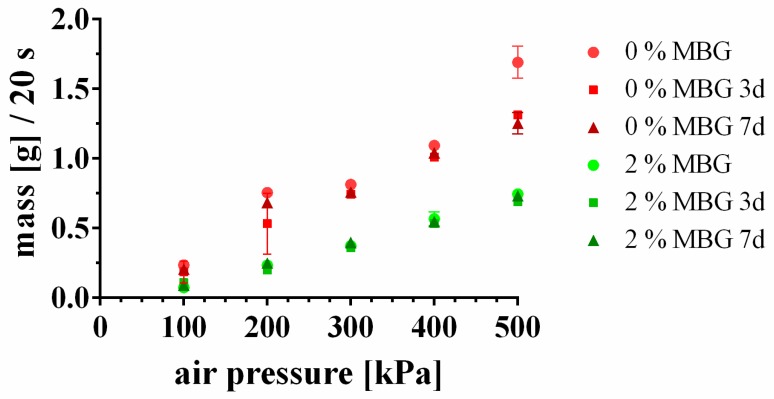
Storability of a pure CPC paste (0%-MBG) and a MBG-CPC composite (2%-MBG-CPC). The extruded mass flow profiles through a 610 µm needle did not change over 7 d, indicating that storability was not impaired by incorporation of MBG. (mean ± standard deviation, n = 3).

Pasty CPC were reported to be storable for years as long as they are not exposed to water (either by immersion or in humid atmosphere) [11,12,13]. To test the influence of MBG on storability, 2 (w/w)% MBG were mixed with the CPC paste and the extrusion profiles of pure CPC paste and MBG-containing CPC paste were measured immediately after mixing and after 3 d and 7 d of storage, respectively. The extrusion profile was identified by determination of the extruded mass within 20 s of extrusion through a 610 µm needle (Figure 1). The extrusion profiles of CPC and 2%-MBG-CPC showed an almost linear slope, indicating that the mass flow increased linearly with increasing air pressure. At all air pressures the mass flow of 2%-MBG-CPC was significantly lower than the mass flow of CPC (*p* < 0.05). Most important, the extrusion profiles after 3 d and 7 d did not change in comparison to freshly mixed pastes, indicating that the storability of MBG-CPC pastes is not impaired by incorporated MBG particles.

### 3.3. Extrudability of MBG-CPC Composite Pastes as a Function of Their Composition

#### 3.3.1. Influence of MBG Particle Concentration on Extrudability of the CPC Paste

**Figure 2:**
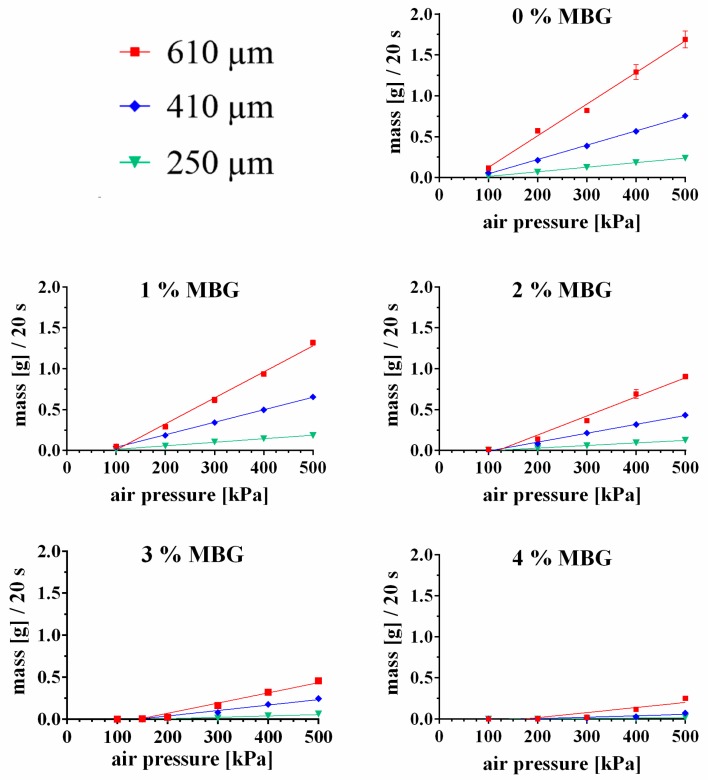
Extrudability, characterized by mass flow through different needle diameters (250 µm—green, 410 µm—blue, 610 µm—red) of CPC pastes with different concentrations of incorporated MBG particles. Higher concentrations of MBG were not possible to extrude. (mean ± standard deviation, n = 3, please consider that most standard deviations are smaller than the data points). R^2^ numbers of the curve fits are shown in Table 3.

The CPC paste was evaluated on its extrusion properties after mixing with MBG particles. A steady and homogeneous extrusion through needles could be observed up to an addition of 4 (w/w)% MBG; higher amounts of MBG led to needle clogging and distinctly inhomogeneous extrusion profiles. The mass flow curves through needles of three different inner diameters (250, 410 and 610 µm) of CPC mixed with 0, 1, 2, 3 and 4 (w/w) % MBG are shown in Figure 2 (shown is the measured mass of released pastes within 20 s extrusion). All extrusion profiles showed a distinct linear behavior, however with increasing amount of MBG and decreasing needle diameters the coefficient of determination (R^2^) decreased, indicating a less homogeneous extrusion through the needle (R^2^ numbers of the extrusion profile fits are shown in Table 3). Moreover, the extruded mass decreased for all needle diameters with increasing MBG concentration; for example the extruded mass (at 500 kPa and a needle diameter of 410 µm) of 0%-MBG-CPC was 1.690 ± 0.10 g and decreased to 0.655 ± 0.01 g (1%-MBG-CPC), 0.433 ± 0.022 g (2%-MBG-CPC), 0.245 ± 0.03 g (3%-MBG-CPC) and 0.073 ± 0.00 g (4%-MBG-CPC), respectively. Remarkably, concentrations of 3% and 4% MBG did not allow extrusion through the 250 µm needle at air pressures <200 kPa, for 4% MBG extrusion was not even possible for the 410 µm needle and air pressures <300 kPa.

#### 3.3.2. Incorporation of Higher Amounts of MBG by Modification of the CPC Paste

Higher concentrations of MBG in MBG-CPC composites should enhance the positive effect on degradation kinetics and release rates of bioactive ions and growth factors, however, higher concentrations of MBS also affect the extrudability of the paste (Figure 2). Two main effects were expected to contribute to mass flow behavior of MBG-CPC pastes and consequently to be adjusting factors for increase of the MBG amount: the MBG particle size and the liquid content of the CPC paste.

The influence of the MBG particle size was investigated by measurements of the mass flow of 2%-MBG-CPC through needles of 250, 410 and 610 µm inner needle diameter (Figure S2). The extrusion profile of MBG of the fraction < 45 µm was not altered in comparison to extrusion profiles with incorporated MBG of all fractions < 180 µm (the amount of particles with an average diameter 45 µm < MBG < 180 µm was > 50%). Surprisingly, this result was independent of the needle diameter (Figure S2). Therefore, it was concluded that the particle size for MBG particles < 180 µm plays a minor role for extrusion of low concentrated MBG-CPC composite pastes. At higher concentrations of MBG, it can be assumed that the role of the particle size will influence the extrusion behavior; however, the tendency of small sized MBG to agglomerate by formation of clusters hinders simple preparation of homogeneous MBG-CPC pastes. Therefore, the fraction < 45 µm was used for following experiments without further decreasing the particle size.

**Figure 3:**
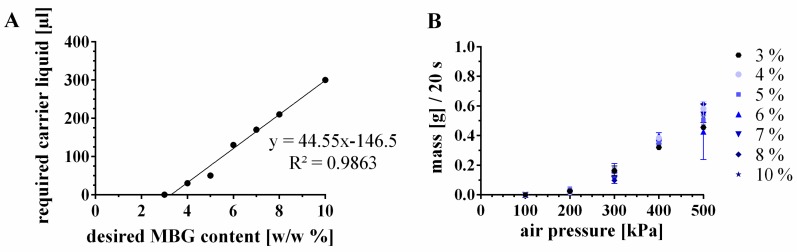
Adjustment of pasty CPC to incorporate MBG particles without impairing the mass flow through a 610 µm needle. (**A**) The mass flow of a 3%-MBG-CPC paste was the blueprint for the mass flows of MBG-CPC composites with higher MBG amounts. The mass flow was adjusted by addition of carrier liquid to the pasty CPC (see Table 1 for the final compositions). (**B**) Extrusion profiles of differently concentrated MBG-CPC pastes with adjusted carrier liquid concentration (mean ± standard deviation, n = 3).

The solid-to-liquid ratio [w/w %] of CPC is 86:14 (Table 1). As a consequence of addition of MBG to the CPC paste, the solid content increased and the ratio was 86.54:13.46 for 4%-MBG-CPC-I, which did not show a controlled and linear mass flow profile. In principle, the addition of more carrier liquid will shift MBG-CPC pastes to a more liquid (pasty) character. Therefore, it was investigated, whether a decrease of the solid-to-liquid ratio towards 86:14 would allow uniform extrusion of MBG-CPC composites with MBG concentrations of ≥4%. Considering that 3%-MBG-CPC demonstrated a controlled extrusion profile, it was determined to mimic the same extrusion profile through a 610 µm needle by addition of carrier liquid (cl) to the composites with higher MBG concentrations. The correlation of the desired MBG content and the required amount of added carrier liquid to the paste, to meet the same mass flow curves, are shown in Figure 3. Until 10 (w/w) % of MBG particles within the CPC paste, a linear relation was observed (Figure 3A, additions of more than 10 (w/w) % were not part of that study, thus a linear relation at higher MBG amounts remains unclear). Interestingly, the liquid content of 6, 7, 8 and 10%-MBG-CPC pastes was higher than 14% (Table 1), but lower amounts of carrier liquid did not meet the intended mass flow curve of 3%-MBG-CPC. The resulting extruded masses of MBG-CPC composites with the adjusted carrier liquid volumes are shown in Figure 3B. No significant changes between the extrusion profiles were observed, proving the correlation between added carrier liquid to the cement for a specific MBG content.

To study the impact of the additional carrier liquid and/or the MBG particles on cement setting in the optimized MBG-CPC composites, the initial setting time was investigated. Recently we have shown the benefit of a setting procedure in water-saturated atmosphere in comparison to setting within an aqueous solution by avoidance of microcracks within 3D plotted strands [16]. Therefore initial setting was characterized by Gilmore needle test using samples placed in water-saturated atmosphere (humidity >95%). As the water has first to penetrate into the matrix by replacing the carrier liquid, this leads to a prolongation of the setting time in comparison to the common experimental setup of mixing the cements with aqueous solution. Pasty CPC had an initial setting time of 153 ± 18 min. CPC + 210 µL carrier liquid (CPC-cl) without added MBG exhibited an initial setting time of 135 ± 12 min. Contrary, 8%-MBG-CPC (including the additional 210 µL carrier liquid) showed a significantly increased initial setting time of 255 ± 21 min (*p* < 0.001). The final setting times could not be detected, however after 3 d of setting in water-saturated atmosphere all three pasty cements hardened to a solid.

### 3.4. Three-Dimensional Plotting of MBG-CPC Composites in Mild Conditions

**Figure 4:**
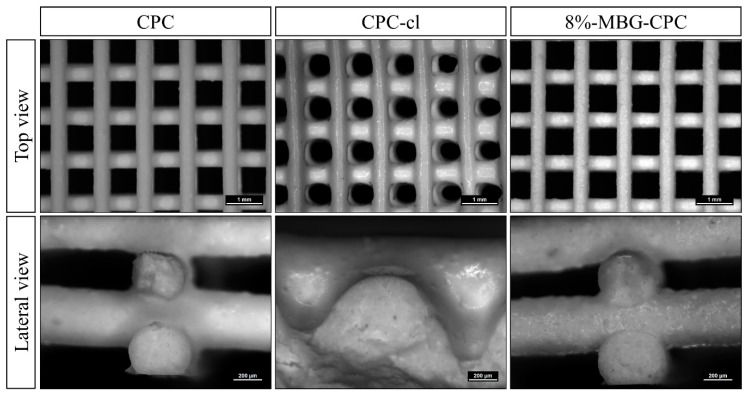
3D plotted scaffolds from unmodified CPC, CPC with an increased amount of carrier liquid (CPC-cl) and addition of 8% MBG particles and the same amount of carrier liquid. Addition of MBG allowed development of lateral pores without distinct changes compared to MBG-free CPC. Scale bars: Top view 1 mm, lateral view 200 µm.

As pasty CPC and its composites with MBG can easily be extruded through a syringe, extrusion-based additive manufacturing can be applied in form of three-dimensional plotting. CPC, CPC-cl (with an added amount of 210 µL carrier liquid) and 8%-MBG-CPC (including 8 w/w % MBG and 210 µL carrier liquid) were plotted with a layer-to-layer orientation of 90° and a strand distance of 1.2 mm. Afterwards the plotted scaffolds were set in water-saturated atmosphere. Stereomicroscopical images of top and lateral views of plotted scaffolds are shown in Figure 4. The shape fidelity of CPC and 8%-MBG-CPC scaffolds was comparable. The plotted strands did not vary in strand width over the whole scaffold area and constituted macropores showed a sharp rectangular structure. In both scaffold types, lateral porosity, and therefore an interconnected pore network throughout the whole scaffold, was achieved. In contrast, CPC-cl scaffolds without MBG showed a less rectangular structure of constituted macropores and the strand widths differed between adjacent layers. This might be caused by a collapse of the plotted strands in z-direction of the scaffold, as seen in the lateral view, therefore open lateral porosity was not achieved and macropores were only connected in z-direction. Scaffold porosity (including macro- and microporosity) was investigated by pycnometry. For CPC and 8%-MBG-CPC, a porosity of 64.7% ± 1.1% and 75.4% ± 5.4%, respectively, was determined, but the difference was not significant. CPC-cl scaffolds revealed a porosity of 41.5% ± 6.4%, which was significantly less compared to those consisting of CPC and 8%-MBG-CPC (*p* < 0.001).

### 3.5. Characterization of Three-Dimensional Plotted MBG-CPC Scaffolds

#### 3.5.1. Mechanical Properties

**Figure 5:**
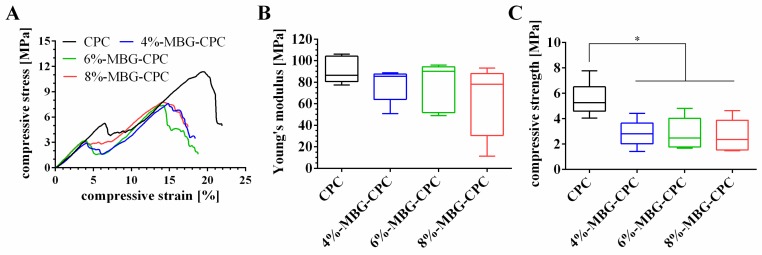
Mechanical properties of CPC, 4%-MBG-CPC, 6%-MBG-CPC and 8%-MBG-CPC scaffolds. (**A**) Representative compressive stress-strain curves. (**B**) Young’s modulus and (**C**) compressive strength determined from the curves (* *p* < 0.05, mean ± standard deviation, n = 5).

Three different MBG-CPC composite pastes were fabricated and plotted to scaffolds: 4%-MBG-CPC, 6%-MBG-CPC and 8%-MBG-CPC (stereomicroscopical images of top and lateral view are shown in Figure S3). After setting and subsequent incubation in deionized water for 7 d, mechanical properties of the scaffolds were investigated by uniaxial compressive tests, MBG-free CPC scaffolds of the same geometry were tested as control. Representative stress-strain curves are shown in Figure 5A. All scaffolds revealed a linear-elastic deformation region which clearly ended by a fraction. Afterwards, all scaffolds showed again increasing compressive stress with increasing strain, until the scaffold fractioned again. Young’s modulus and compressive strength were analysed from the first linear region (Figure 5B,C). All scaffolds showed no significant difference for the Young’s modulus (e. g., 76.5 ± 22 MPa for 8%-MBG-CPC and 91.2 ± 12 MPa for CPC). Though, the compressive strength of CPC (5.5 ± 1.4 MPa) was significant higher compared to the compressive strengths of MBG-CPC scaffolds (approximately 2.8 MPa for all compositions). For the tested composites, the amount of MBG particles within the CPC did neither influence Young’s modulus nor compressive strength significantly.

#### 3.5.2. Microstructure

**Figure 6:**
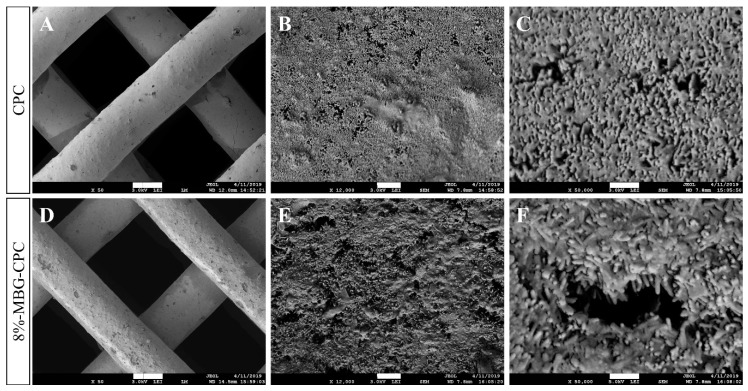
SEM images for 3D plotted scaffolds from unmodified CPC (**A**–**C**) and an 8%-MBG-CPC composite (**D**–**F**). The incorporation of MBG led to no distinct changes regarding surface texture or cristallite formation. (scale bars (**A**,**D**): 300 µm; (**B**,**E**): 1 µm; (**C**,**F**): 300 nm).

To investigate the microstructure of the MBG-CPC composite in comparison to CPC, scaffolds of CPC and an 8%-MBG-CPC composite were plotted, set in water-saturated atmosphere for 3 d und incubated in water for 7 d. Although previous results showed a significant increase for initial setting times in the presence of MBG, no macroscopic difference was observed after setting. SEM images (Figure 6) for microstructure analysis verified this. No differences between CPC and the composite in surface texture are seen in lower magnification (Figure 6A,D). Additionally, higher magnifications (Figure 6B,C,E,F) show the same crystallite structure for both materials, demonstrating that the incorporation of MBG in CPC did not impede the CHDA formation.

### 3.6. Ion Release from Sr-MBG-CPC Scaffolds

Calcium containing MBG can be modified in their atomic composition by (even partly) substitution of the calcium precursor (CaNO_3_) with precursors containing other metal ions. It can be expected, that the composition of the MBG will influence the release profile of those ions out of MBG-CPC composites. To prove that system, Sr^2+^ was used as model ion. MBG containing 0 (mol/mol_MBG_) % Sr and 15% Ca (0SrMBG), 5% Sr and 10% Ca (5SrMBG), 10% Sr and 5% Ca (10SrMBG) and finally 15% Sr and 0% Ca (15SrMBG) were produced. Fabrication and grinding, as well as particle size distribution were not affected by the substitution of the precursor. Afterwards, 8 (w/w) % of the different MBG particles were mixed with CPC and scaffolds were fabricated by 3D plotting. The plotting parameters were not affected by different MBG particles, thus scaffolds of almost the same size and volume could be fabricated. After setting in water-saturated atmosphere, scaffolds were weighed and incubated in PBS over 21 d with a complete exchange of the supernatants every 3–4 d. For the 8%-15SrMBG-CPC composite, the influence of macropores, enhancing the surface to volume ratio, on ion release was tested by comparison of a plotted structure to a bulk control of same mass (see Figure S4).

**Figure 7:**
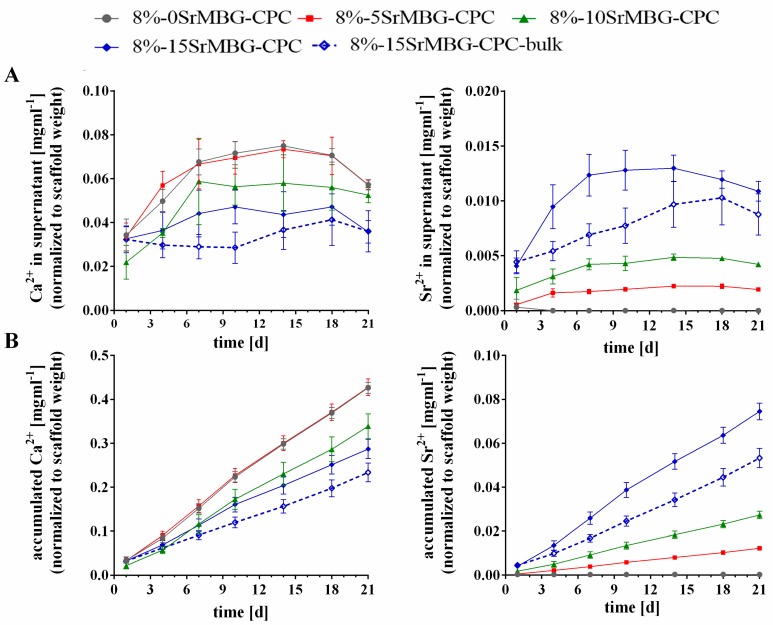
Release of Ca^2+^ and Sr^2+^ from 8%-MBG-CPC scaffolds containing MBG particles with different Sr content (0–15%) into PBS solution, quantified by ICP-OES. The determined concentrations were normalized to the weight of each sample before the incubation in PBS. (**A**) Measured release within the supernatants, (**B**) accumulated release curves (mean ± standard deviation, n = 6).

The concentrations (normalized to the starting weight of the scaffolds) of Ca^2+^ and Sr^2+^ ions in the supernatants are shown in Figure 7A. The Ca^2+^ content was the highest for 8%-0SrMBG-CPC and 8%-5SrMBG-CPC with only negligible differences between the groups. As expected, the lowest concentrations of Ca^2+^ were found for 8%-15SrMBG-CPC and its bulk control, noticeably with more measured Ca^2+^ at early time points released by the plotted scaffold. Sr^2+^ was not measured in Sr-free 8%-0SrMBG-CPC scaffolds. With increasing Sr^2+^ content in the MBG, more Sr^2+^ was released from the SrMBG-CPC composites. Highest Sr^2+^ concentrations in the supernatant were found for 8%-15SrMBG-CPC composites, but the plotted structures released between 1 d and 18 d significantly (*p* < 0.001) more Sr^2+^ compared to the bulk. The accumulated release of Ca^2+^ and Sr^2+^ is shown in Figure 7B. The curves show that both ions were released in a sustained manner indicating a constant release from the scaffolds.

## 4. Discussion

CaP materials are accepted as outstanding bone substitutes in orthopedic and maxillofacial surgery. To treat large osseous defects, injectable CPC formulations were developed, which can be applied easily in clinical applications [24,25]. Recently, a non-aqueous cl-based CPC with a specific solid-to-liquid ratio of 86:14 showed good injectability and prevented phase separation in the needle tip (first results of a 85:15 composition of the same cement system were optimized over time) [12,23]. Additionally, it possesses a long-term storability and therefore maintains its ready-to-use character until contact with aqueous media. Therefore, this CPC is a highly suitable material for 3D plotting and was used as a composite component in this study.

Due to the incorporation of another solid phase in the form of MBG, we demonstrated the expected change of the injectability (by studying the extrusion profile through plotting needles) of the CPC-based composite depending on the MBG amount. Without any further modifications, composites with up to 4 (w/w) % were extrudable. As a consequence, additional changes throughout the composite development were required and performed to achieve higher MBG amounts and maintain the favorable extrusion properties of the CPC at the same time. Schumacher et al. showed the incorporation of up to 10 wt % MBG in the solid phase for a MBG-CPC composite [19], while Li et al. added 20 wt % MBG during paste preparation [8]. Both approaches were based on an aqueous powder-liquid system. In that case, the MBG is added to the cement powder. By adjusting the amount of the liquid phase, processible pastes were obtained. These composites are only partially comparable to the MBG-CPC composites developed in this study. Immediately after mixing the components, the cement setting reaction started, resulting in a limited application time, which is practically unlimited for the cl-based MBG-CPC composite pastes. Nevertheless, similarly to the named studies, the maintenance of a solid-to-carrier liquid-ratio of approximately 86:14 led to processible MBG-CPC pastes (Table 1). By addition of carrier liquid to MBG-CPC composites with a MBG content >3%, injectability, and therefore the extrusion profile, was maintained until 10 (w/w) % (which complies to approximately 10.4 wt % MBG in the solid phase); however, higher amounts of MBG should also be possible.

While extrudability and storability was maintained, the addition of both MBG and carrier liquid resulted in altered initial setting times. The slightly reduced initial setting time in the case of CPC-cl comparing to pure CPC might probably be an effect of the reduced amount of precursor in the same sample volume. Due to the addition of MBG, the initial setting time increased by 66%. For powder–liquid systems, a similar effect was observed [8,9,19]. The reason for the extended setting time remains unclear at this point. A possible explanation was shown for a CPC–calcium silicate system [26]. The high water absorption capacity of the calcium silicate affected the hydration of the calcium phosphate phase and the diffusion path for ions between single CaP crystal grains was prolonged because of the additional silicate phase [26]. A similar explanation is conceivable for the MBG-CPC composites. The MBG particles could retard the transformation of the α-TCP. Although a closer examination of this effect is necessary, MBG-CPC composites hardened in water-saturated atmosphere after 3 days, similarly as achieved for pure CPC [16].

The character of good injectability of the highly viscous MBG-CPC composite pastes makes them interesting for automated dispensing processes, especially in low-temperature extrusion-based additive manufacturing, namely 3D plotting. The usage of additive manufacturing techniques for scaffold fabrication opens up various possibilities to improve tissue defect healing. Not only are individual geometries producible, the integration of macroporosity is beneficial for cell and tissue ingrowth, improved biomaterial resorption and therefore better tissue regeneration. 3D plotting of MBG-free CPC formulations were intensely studied before, demonstrating advantageous properties of three-dimensional CaP scaffolds for bone tissue engineering and treatment of bone defects [7,13,14,15,27,28]. In addition, pure bioglass-based scaffolds with similar, but not identical, glass compositions, could be fabricated by blending particles with carboxymethyl cellulose, which vanished in a subsequent sintering step [29]. In such a pure bioactive glass scaffold, further functionalization by growth factors is only possible after post-processing. Therefore, several groups developed bioactive glass-based composites that allow the fabrication of scaffolds and their post-processing in mild conditions which do not impair loaded drugs or proteins.

For example, bioactive glasses were successfully incorporated into hydrogels like PVA, alginate or alginate dialdehyde-gelatin, which can be post-processed by ionic or mild thermal crosslinking [30,31,32]. In the approach investigated here, MBG particles will contribute to the CDHA scaffold by enhanced degradation and with a sustained growth factor release from the composite material. In combination with multichannel plotting, it will be possible to produce MBG-CPC scaffolds releasing multiple growth factors in a very specified spatiotemporal manner, similarly as shown before for hydrogel-CPC composite scaffolds [17]. To the best knowledge of the authors, the first approach of extrusion-based fabrication of MBG-CPC scaffolds was performed by Li et al. [8]. The fabricated structures showed a high shape fidelity, however the extrusion time was limited to 120 min due to setting processes within the cementitious composite already before and during extrusion. Interestingly, only the addition of bioactive glass particles allowed the extrusion within an appropriate time window, whereas MBG-free scaffolds were not plottable [8]. In contrast, the cl-based MBG-CPC composite developed here, could be processed without remarkable changes after 7 d; plotting after longer storage period has not been tested but is expected. This allows the fabrication of complex shaped and/or large CaP constructs, which require long plotting times [33]. Furthermore, we could show that MBG-CPC composites with various MBG content can be produced, which will impact degradation properties of the composite material [19], while maintaining the extrudability and therefore plottability. Mechanical characterization of plotted scaffolds showed no difference between CPC and various MBG-CPC composites in terms of Young’s modulus and a decrease in compressive strength in the case of the composites. Microstructure analysis showed no difference regarding the crystallite formation and therefore the change in mechanical properties for composites is probably traced back to the incorporation of the glass particles and the different mechanical properties of the MBG itself. Li et al. showed a similar effect for their composite system [8].

During degradation, MBG release ions, which impact bone metabolism, like silicon, calcium and phosphorus [34,35]. By modification of the bioactive glass composition, also other ions can be released, among many others, divalent ions like magnesium and copper or trivalent ions like cerium [36,37]. In the investigated approach, the calcium content was stepwise substituted by strontium; Sr^2+^ ions were demonstrated to accelerate osteogenic differentiation but decelerate osteoclastic resorption [38,39,40,41]. The investigated ions were Ca^2+^, released by MBG and CDHA, as well as Sr^2+^, released only by MBG. This caused a 10-fold higher release of calcium ions than strontium ions from the MBG-CPC composite scaffolds, however the released levels of strontium ions were in the range which was shown to impact bone metabolism. Schumacher et al. showed a positive effect on proliferation and osteogenic differentiation of osteoblastic progenitor cells for strontium ion concentrations of 0.001–1 mM [42] and a reduced osteoclastic resorption without inhibition of osteoclastogenesis for 0.01–1 mM [43]. Both, calcium and strontium ions were released into PBS in a sustained manner and dependent on their molar content within the MBG. Interestingly, the measured calcium ion levels of 8%-0SrMBG-CPC and 8%-5SrMBG-CPC were similar. Most probably, the amount of calcium ions released by the CDHA phase was the same, therefore the calcium ion release of both MBG types must be considered as equal. One possible explanation for these findings might be that silica-based MBG containing 10 mol % Ca develop a bicontinuous 3D cubic mesoporous channel system which evolves to a 2D hexagonal for higher Ca content [44]. Therefore, the former allows easier fluid accessibility resulting in a comparatively better ion release. A different explanation could be that due to the larger size of the Sr^2+^ cation compared to Ca^2+^ the glass network expands, which results in a weakening of the glass network. Fredholm et al. showed the significance of this effect for different Sr substitutions for Ca [45]. Furthermore, they showed an increased degradation of Sr containing bioglasses, resulting in an improved ion release [46].

Moreover, it could be demonstrated, that significantly more strontium ions were released from an 8%-15Sr-MBG-CPC scaffold which was plotted in comparison to a bulk structure. Most probably, the increased surface area (theoretically 7.7 cm² for plotted scaffolds in comparison to 3.1 cm² for bulk samples) of a plotted scaffold with macropores and convexly shaped strands contributes positively to the degradation of both MBG and CPC The increased levels of strontium ions are an indicator that the delivery of biological active substances, like such ions, but in principle also loaded growth factors, can be tailored in 3D plotted scaffolds, e.g., by changing the strand diameter and therefore the surface area of the scaffold. In addition, the amount of MBG and, as shown in this work, its composition influence the release of biologically active substances, making the plottable MBG-CPC composite material a highly flexible system allowing the release of ions and growth factors to be tailored to individual patient requirements.

## 5. Conclusions

Herein, we investigated composites consisting of a self-setting calcium phosphate cement and mesoporous bioactive glasses (MBG-CPC) for the treatment of bone defects. By adjusting the solid-to-liquid ratio, MBG contents could be achieved in the range of 0–10%; however, even higher contents will be possible without decreasing the injectable character of MBG-CPC pastes. Afterwards, MBG-CPC scaffolds were successfully fabricated by three-dimensional plotting. Macroporous, plotted scaffolds showed a higher release of biological active degradation products like calcium and strontium ions compared to bulk samples, indicating an improved degradation due to the increased surface area. Therefore, the developed MBG-CPC system can contribute in two ways to patient-individual therapies: degradable scaffolds can be additively manufactured in a shape required for a specific defect and the release of biological active substances can be tailored to patient-individual doses.

## Figures and Tables

**Table 1:** MBG-CPC composites of different concentrations investigated in this study.

MBG	Molar ratio	Label	CPC	MBG	Additional Carrier Liquid			Composition	
[w_MBG_/w_CPC_ %]	[80 Si/X/5 P]	-	[g]	[g]	[µL]	[g]	[w_cl_/w_CPC_ %]	Solid [w/w %]	Liquid [w/w %]
0	-	CPC	10	0	0	0	0	86	14
	-	CPC-cl	10	0	210	0.189	1.89	84.4047	15.5952
1	15 Ca^2+^	1%-MBG-CPC	10	0.1	0	0	0	86.1386	13.8613
2	15 Ca^2+^	2%-MBG-CPC	10	0.2	0	0	0	86.2745	13.7254
3	15 Ca^2+^	3%-MBG-CPC	10	0.3	0	0	0	86.4077	13.5922
4	15 Ca^2+^	4%-MBG-CPC-I	10	0.4	0	0	0	86.5384	13.4615
	15 Ca^2+^	4%-MBG-CPC	10	0.4	30	0.027	0.27	86.3143	13.6856
5	15 Ca^2+^	5%-MBG-CPC	10	0.5	50	0.045	0.45	86.2968	13.7031
6	15 Ca^2+^	6%-MBG-CPC	10	0.6	130	0.117	1.17	85.8449	14.1550
7	15 Ca^2+^	7%-MBG-CPC	10	0.7	170	0.153	1.53	85.6905	14.3094
8	15 Ca^2+^	8%-MBG-CPC/ 8%-0SrMBG-CPC	10	0.8	210	0.189	1.89	85.5400	14.4599
	10 Ca^2+^, 5 Sr^2+^	8%-5SrMBG-CPC	10	0.8	210	0.189	1.89	85.5400	14.4599
	5 Ca^2+^, 10 Sr^2+^	8%-10SrMBG-CPC	10	0.8	210	0.189	1.89	85.5400	14.4599
	0 Ca^2+^, 15 Sr^2+^	8%-15SrMBG-CPC	10	0.8	210	0.189	1.89	85.5400	14.4599
10	15 Ca^2+^	10%-MBG-CPC	10	1.0	300	0.27	2.7	85.1818	14.8181

**Table 2:** Plotting parameters for different compositions.

Label	Pressure [kPa]	Plotting Speed [mms^−1^]
CPC	120	5.5
CPC-cl	50	10
8%-MBG-CPC	350	10
8%-xSrMBG-CPC (x = 0, 5, 10, 15)	325	10

**Table 3:** Coefficients of determination of the linear fitted mass flow profiles dependent on different needle types (Figure 2).

R^2^	610 µm	410 µm	250 µm
0% MBG	0.9931	0.9987	0.9994
1% MBG	0.9942	0.9995	0.9988
2% MBG	0.9807	0.9946	0.9914
3% MBG	0.9664	0.9631	0.9136
4% MBG	0.8163	0.7998	0.795

## Supplementary Materials

The following are available online at https://www.mdpi.com/1996-1944/12/12/2022/s1: Figure S1: Particle size distribution of MBG particles. Figure S2: Influence of MBG particle size on extrusion behavior in needles with inner diameters of 610 μm, 410 μm and 250 μm. Figure S3: Stereomicroscopical images of three-dimensional plotted CPC and 4%-, 6%- and 8%-MBG-CPC composite scaffolds. Figure S4: Mass distribution of the plotted and bulk scaffolds investigated in Sr and Ca release experiments.
