# Supplementary material for: Development and Characterization of Composites Consisting of Calcium Phosphate Cements and Mesoporous Bioactive Glass for Extrusion-Based Fabrication
Source: Materials (Basel). 2019 Jun 24;12(12):2022. doi: 10.3390/ma12122022 (PMC6630970; doi:10.3390/ma12122022)
Supplement: Supplementary file 1 [file materials-12-02022-s001.pdf]

materials

Supplementary Materials

Development and Characterization of Composites Consisting of Calcium Phosphate Cements and Mesoporous Bioactive Glass for Extrusion-Based Fabrication

Richard Frank Richter, Tilman Ahlfeld, Michael Gelinsky * and Anja Lode *

Centre for Translational Bone, Joint and Soft Tissue Research, University Hospital Carl Gustav Carus and Faculty of Medicine, Technische Universität Dresden, 01307 Dresden, Germany; richard.f@rfrport.net (R.F.R.); tilman.ahlfeld@tu-dresden.de (T.A.)

* Correspondence: michael.gelinsky@tu-dresden.de (M.G.); anja.lode@tu-dresden.de (A.L.); Tel.: +49-351-458-6694 (M.G.); +49-351-458-6692 (A.L.).

Characterization of MBG particle size and influence on plottability

A batch of MBG particles were synthesized as described previously.[1] After the calcination process, the particles demonstrated a wide particle size distribution (**Figure S1A, red**). Therefore, manually grinding was applied to the batch, resulting in a distinct decrease of the average particle size, however more than 50 % of the batch displayed a particle size between 45 and 180 µm (**Figure S1A, yellow**). After an additional grinding of each fraction, more than 99 % of the entire batch was identified as smaller than 45 µm (**Figure S1A, green**). This fraction tended to agglomerate as exposed to vibration (**Figure 1SB**, red arrows) and therefore could not be downsized further by sieve fractioning.

The particle size distribution might influence the injectability/extrusion profile of a MBG-CPC composite by agglomeration of larger particles within the tip of the needle. The influence of particle size distribution on the mass flow was investigated utilizing a 2%-MBG-CPC paste (**Figure S2**). At this amount of added MBG, no significant change in the extruded mass could be detected, independent of the used needle size. However, for higher amounts of MBG, a particle agglomeration seems likely to happen at the tip of the needle, therefore only particles displaying particles sizes <45 µm were used for investigation.

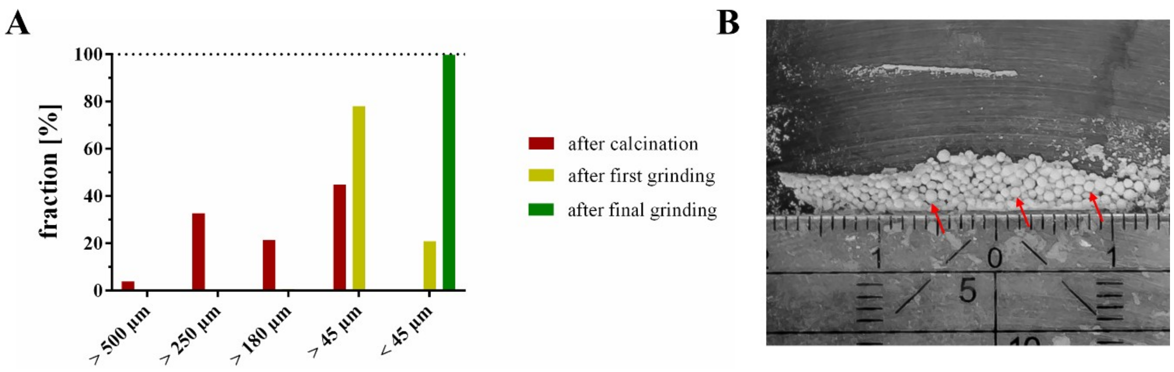

Figure S1. Particle size distribution of MBG particles. The calcified MBG particles were thoroughly grinded until more than 99 % of the batch could be identified as smaller than 45 µm.

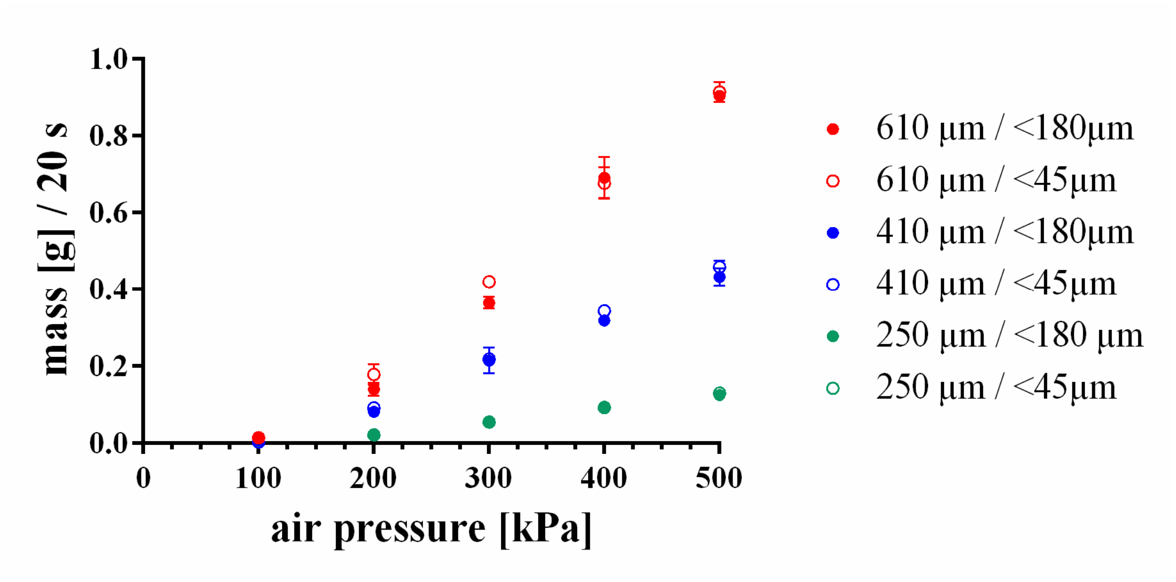

Figure S2. Influence of MBG particle size on extrusion behavior in needles with inner diameters of 610 µm, 410 µm and 250 µm. Filled circles represent the extruded mass of a 2%-MBG-CPC composite paste with MBG particles which were grinded only one time, open circles represent the extruded mass of a 2%-MBG-CPC composite including MBG after the final grinding procedure (n=3, mean±SD, please consider that most SD are smaller than the data points).

3D plotting of MBG-CPC composites

MBG-CPC composite pastes containing MBG contents >3 % were developed to reveal the same extrusion profile (Figure 3). Against this background, it is expected that the plottability of these composites is the same. The plotting parameters of 4%, 6% and 8%-MBG-CPC composite pastes differed only negligibly. Scaffolds with 10 layers were plotted and pure CPC scaffolds were taken as control. For both vertical and lateral porosity, no distinct changes within plotted scaffolds were observed, independent of the amount of MBG (**Figure S3**), implying the same pre-and post- extrusion behavior of the pastes.

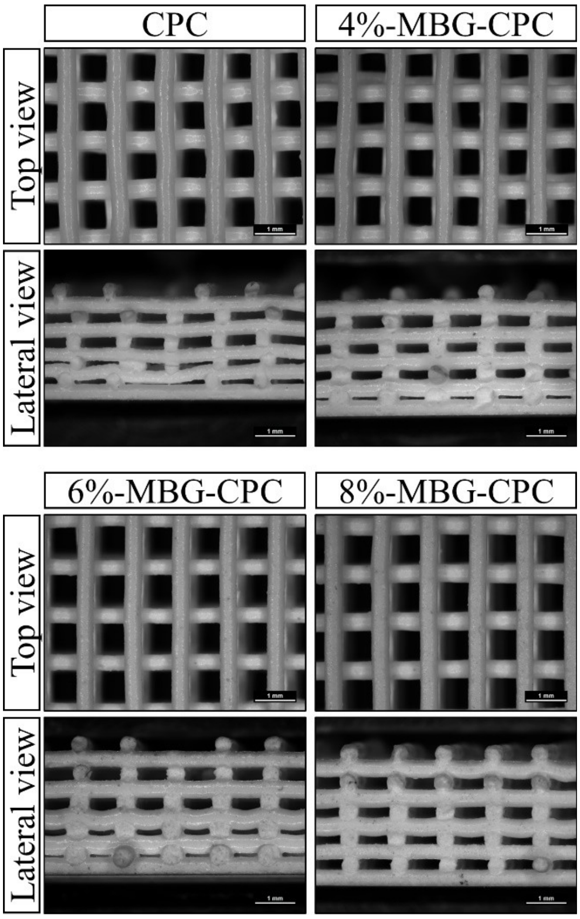

Figure S3. Stereomicroscopical images of three-dimensional plotted CPC and 4%-,6%- and 8%-MBG-CPC composite scaffolds. All scaffolds revealed an interconnected pore network, provoked by open pores in vertical and lateral direction. All scaffolds revealed no distinct macroscopic differences of the plotted structures.

Mass distribution of plotted 8%-MBG-CPC pastes for evaluation of calcium and strontium release

The plotted scaffolds used for ion release investigations were set and weighed. Bulk controls were fabricated with the intension to meet the same mass. However, very slight differences could not be prevented (**Figure S4**). As a higher mass of a MBG-CPC composite is likely going to release more ions, it was decided to normalize the released ion concentrations to the initial scaffold weights.

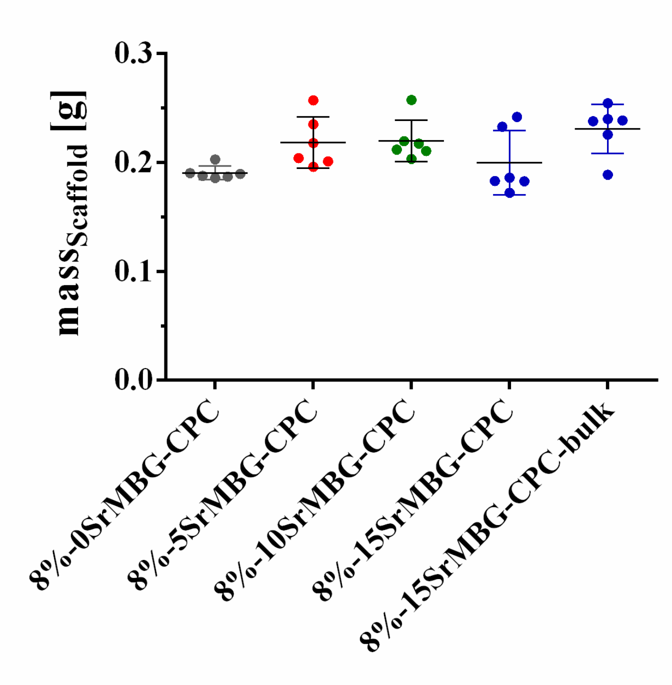

Figure S4. Mass distribution of the plotted and bulk scaffolds investigated in Sr and Ca release experiments.

References

1. Yan, X.; Yu, C.; Zhou, X.; Tang, J.; Zhao, D. Highly Ordered Mesoporous Bioactive Glasses with Superior In Vitro Bone-Forming Bioactivities. *Angew. Chem. Int. Ed.* **2004**, *43*, 5980–5984.

© 2019 by the authors. Submitted for possible open access publication under the terms and conditions of the Creative Commons Attribution (CC BY) license (http://creativecommons.org/licenses/by/4.0/).
